# Preferences for onward health data use in the electronic age among maternity patients and providers in South Africa: a qualitative study

**DOI:** 10.1080/26410397.2023.2274667

**Published:** 2023-11-20

**Authors:** Amnesty LeFevre, Olivia Welte, Kearabetswe Moopelo, Nicki Tiffin, Gaolatlhe Mothoagae, Nobukhosi Ncube, Nasiphi Gwiji, Manape Shogole, Amy L. Slogrove, Nomakhawuta Moshani, Andrew Boulle, Jane Goudge, Frances Griffiths, Lee Fairlie, Ushma Mehta, Kerry Scott, Nirvana Pillay

**Affiliations:** a Associate Professor, School of Public Health, University of Cape Town, Falmouth Rd, Observatory, Cape Town 7925, South Africa.; b Social Scientist, School of Public Health and Family Medicine, University of Cape Town, Cape Town, South Africa; c Social Scientist, Sarraounia Public Health Trust, Johannesburg, South Africa; d Professor, South African Bioinformatics Institute, Life Sciences Building, University of the Western Cape, Bellville; e Associate Researcher, Sarraounia Public Health Trust, Johannesburg, South Africa; f Social Scientist, Sarraounia Public Health Trust, Johannesburg, South Africa; g School of Public Health and Family Medicine, University of Cape Town, Cape Town, South Africa; h Social Scientist, Sarraounia Public Health Trust, Johannesburg, South Africa; i Associate Professor, Faculty of Medicine and Health Sciences, Department of Paediatrics & Child Health, Stellenbosch University, Worcester, South Africa; j Social Scientist, School of Public Health, University of Cape Town, Cape Town, South Africa; k Professor, School of Public Health, University of Cape Town, Cape Town, South Africa; l Professor, Centre for Health Policy, School of Public Health, University of the Witwatersrand, Johannesburg, South Africa; m Professor, Warwick Medical School, Warwick, UK; Professor, Centre for Health Policy, School of Public Health, University of the Witwatersrand, Johannesburg, South Africa; n Director of Maternal and Child Health, Wits RHI, Faculty of Health Sciences, University of the Witwatersrand, Johannesburg, South Africa; o Associate Professor, School of Public Health, University of Cape Town, Cape Town, South Africa; p Independent research consultant, Toronto, Canada; Associate Faculty, Johns Hopkins School of Public Health, Baltimore, USA; q Director, Sarraounia Public Health Trust, Johannesburg; Visiting Researcher, School of Sociology, University of the Witwatersrand, Johannesburg, South Africa

**Keywords:** informed consent, South Africa, electronic data, digital health, maternal health

## Abstract

Despite the expanding digitisation of individual health data, informed consent for the collection and use of health data is seldom explicitly sought in public sector clinics in South Africa. This study aims to identify perceptions of informed consent practices for health data capture, access, and use in Gauteng and the Western Cape provinces of South Africa. Data collection from September to December 2021 included in-depth interviews with healthcare providers (*n* = 12) and women (*n* = 62) attending maternity services. Study findings suggest that most patients were not aware that their data were being used for purposes beyond the individualised provision of medical care. Understanding the concept of anonymised use of electronic health data was at times challenging for patients who understood their data in the limited context of paper-based folders and booklets. When asked about preferences for electronic data, patients overwhelmingly were in favour of digitisation. They viewed electronic access to their health data as facilitating rapid and continuous access to health information. Patients were additionally asked about preferences, including delivery of health information, onward health data use, and recontacting. Understanding of these use cases varied and was often challenging to convey to participants who understood their health data in the context of information inputted into their paper folders. Future systems need to be established to collect informed consent for onward health data use. In light of perceived ties to the care received, these systems need to ensure that patient preferences do not impede the content nor quality of care received.

## Résumé

Malgré la numérisation croissante des données de santé individuelles, le consentement éclairé pour la collecte et l’utilisation des données de santé est rarement explicitement demandé dans les dispensaires du secteur public en Afrique du Sud. Cette étude vise à identifier les perceptions des pratiques de consentement éclairé pour la capture de données de santé, l’accès à ces données et leur utilisation dans les provinces du Gauteng et du Cap-Occidental en Afrique du Sud. Le recueil de données de septembre à décembre 2021 comprenait des entretiens approfondis avec des prestataires de soins de santé (*n* = 12) et des femmes (*n* = 62) s’étant rendues dans des services de maternité. Les résultats de l’étude indiquent que la plupart des patientes ne savaient pas que leurs données étaient utilisées à des fins allant au-delà de la fourniture individualisée de soins médicaux. Comprendre le concept d’utilisation anonymisée des données de santé électroniques s’est parfois révélé difficile pour les patientes qui concevaient leurs données dans le contexte limité de dossiers et de livrets papier. Interrogées sur leurs préférences en matière de données électroniques, les patientes se sont massivement prononcées en faveur de la numérisation. Elles jugeaient que l’accès électronique à leurs données de santé pouvait faciliter un accès rapide et suivi aux informations sanitaires. Les patientes ont également dû donner leurs préférences, notamment concernant la fourniture d’informations sanitaires, l’utilisation ultérieure de données de santé et la reprise de contact. La compréhension de ces cas d’utilisation variait et était souvent difficile à transmettre aux participantes qui comprenaient leurs données de santé dans le contexte des informations saisies dans leurs dossiers papier. De futurs systèmes doivent être mis en place afin de recueillir le consentement éclairé pour une utilisation ultérieure des données de santé. À la lumière des liens perçus avec les soins reçus, ces systèmes doivent garantir que les préférences des patients n’entravent pas le contenu ni la qualité des soins reçus.

## Resumen

A pesar de la creciente digitalización de datos de salud individuales, rara vez se busca explícitamente el consentimiento informado para la recolección y el uso de esos datos en los centros de salud pública de Sudáfrica. Este estudio pretende identificar las percepciones de las prácticas de consentimiento informado para la captura, la accesibilidad y el uso de datos de salud en las provincias de Gauteng y del Cabo Occidental de Sudáfrica. La recolección de datos realizada entre septiembre y diciembre de 2021 consistió en entrevistas a profundidad con prestadores de servicios de salud (*n* = 12) y con mujeres (*n* = 62) que asistieron a servicios de maternidad. Los hallazgos del estudio indican que la mayoría de las pacientes no eran conscientes de que sus datos se estaban utilizando para fines más allá de la prestación de atención médica individualizada. En ocasiones, a las pacientes que entendían sus datos en el contexto limitado de expedientes y folletos impresos les resultaba difícil comprender el concepto de uso anónimo de datos de salud electrónicos. Ante la interrogante sobre sus preferencias de datos electrónicos, las pacientes estaban abrumadoramente a favor de la digitalización y consideraban el acceso electrónico a sus datos de salud como algo que facilita el acceso rápido y continuo a la información sobre salud. Además, se les preguntó sobre sus preferencias respecto a la entrega de información sobre salud, el uso de datos de salud en adelante y el contacto adicional. La comprensión de estos casos de uso variaba y a menudo resultaba difícil transmitirla a las participantes que entendían sus datos de salud en el contexto de información ingresada en sus expedientes impresos. Es necesario establecer futuros sistemas para obtener el consentimiento informado para el uso de datos de salud en adelante. En vista de los vínculos percibidos con la atención recibida, estos sistemas deben garantizar que las preferencias de las pacientes no impidan ni el contenido ni la calidad de la atención recibida.

## Introduction

Health systems are moving rapidly towards the increasing digitisation of individual health data, including sensitive personal information on health status, clinical diagnoses, and laboratory test results.^1^ The expanding digitisation of health information has occurred concurrently with near ubiquitous access to mobile phones^2^ and a shift towards remote care management which aims to facilitate clinically driven, remote monitoring, care, and education of patients.^3^ The combination of sensitive health data and the use of digital platforms well suited to replication and dissemination has led to an increase in the risk of data misuse or breaches.^4^

Emerging legislation in a number of countries has sought to protect personal information by establishing standards and codes of conduct for data processing and flow.^5^ In 2021, South Africa enacted the Protection of Personal Information Act (POPIA), which sets out the legal conditions for processing personal information in order to protect people from financial crime and identity theft, and secure the right to privacy. POPIA follows the Promotion of Access to Information Act (PAIA) of 2000 which enshrines the constitutional right for individuals to be able to access and review data held by any public or private entity, on request.^6^ POPIA and PAIA build onto South Africa’s National Health Act of 2003, which permits the use of de-identified data, assuming individuals cannot be re-identified, by or on behalf of a public body^7^ – including the Department of Health. Ensuring compliance with POPIA, PAIA and the Health Act requires suitable logging and storage of data, as well as systems for conveying to individuals what data has been collected on them, how it is being used, and by whom.

Consent refers to a “person’s expression of agreement with a proposed action”.^8^ In the context of public sector health services, consent for the collection and use of data is seldom explicitly sought outside of clinical trials. In rare instances where health care client consent is solicited during the provision of routine health care, language used is often opaque,^4^ processes for ascertaining beneficiary competency or understanding are not articulated, and the request for consent is administered by the providers who are simultaneously providing health services.

Traditionally in South Africa, clinical record-keeping by health care providers has been constrained to paper-based folders and disease or health area booklets including the Road to Health card for children and the Maternity Case Record for pregnant and postpartum women. These booklets may be the only personal health data patients have access to. Increasingly, however, paper-based records are being complimented by a number of electronic registries and databases routinely used in hospitals and primary health care clinics which capture varied types of personal health data, and the National Health Laboratory Services (NHLS) capture personal health information pertaining to laboratory testing undertaken for government health care clients. In addition, an increasing number of digital health programmes capture personal or health information including the referral mobile application VULA, direct to beneficiary mobile health programme MomConnect, and a range of frontline health worker solutions (CommCare, DHIS2, Catch, and Match). In some provinces, including the Western Cape, data from multiple sources are integrated into a central health information exchange which serves as a centralised data repository.^9^

In this paper, we aim to identify perceptions of consent practices for health data capture, access, and use in Gauteng and the Western Cape provinces of South Africa. Data collection occurred through in-depth interviews with health care providers and women attending maternity services in Western Cape and Gauteng Provinces.

## Methods

### Conceptualising consent

**Figure 1.**
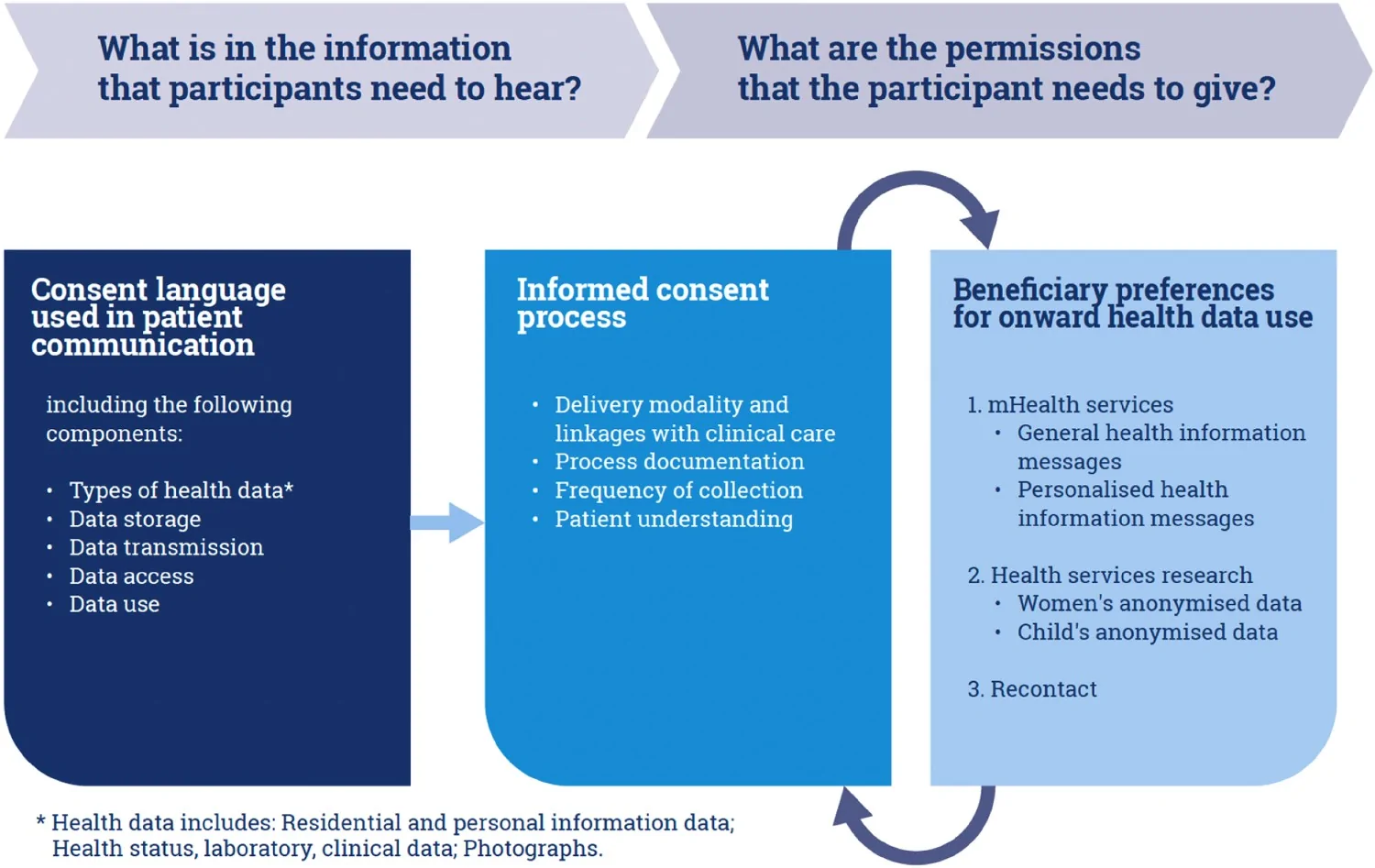
Conceptualising processes for requesting informed consent for onward health data use outside of personalised care

In this paper, we focus on perceptions about onward health data collection and use outside of personalised delivery of health care services. Three different types of health-related data are considered: residential and personal information (not directly health-related, inclusive of residential address and phone number); health status (vital statistics, clinical, and laboratory data); and clinically relevant photographs. In Figure 1, we distinguish between the information that participants need to hear and the permissions that the participant needs to give. The information participants need to hear is represented by the consent language and informed consent process. The permissions that participants need to give for onward health data use is related to illustrative data use cases for: 1. Mobile health services, 2. Health services research, and 3. Recontact for future, not yet defined, research. Two examples of mobile health services were included: receipt of generalised health information messages and personalised health information messages, including appointment alerts and reminders. Health services research included the use of women and child anonymised health data.

Respondents were asked first about their understanding of the word “consent”. They were then asked about current processes for capturing consent for varied types of health data, and preferences for onward health data use, accessing health data. We close by presenting respondent recommendations on how to improve consent capture for onward health data going forward.

### Setting

This study was conducted within three study sites of the UBOMI BUHLE (Understanding Birth Outcomes from Mothers and Infants, Building Healthcare by Linking Exposures) project involving the implementation of a national pregnancy exposure registry (NPER).^24^ The sample was drawn from midwife obstetric units (MOUs) in two clinics in the Western Cape (urban and peri-urban) and one in Gauteng (urban).

In the Western Cape, the urban health facility selected is located 15 km from Cape Town and falls under the Nyanga Health District of the Metro Region. The clinic provides primary health care services to a population of over 100,000 predominately isiXhosa speaking (89%) Black Africans (99%).^10^ This densely populated area (15,000/km^2^) is characterised by high levels of violence and poverty, as well as limited water and sanitation.^10^ Findings from the 2011 census suggest that 40% of the labour force is unemployed; 71% of households have a monthly income of R3,200 (US$230) or less; 48% of households are considered “informal” dwellings; 37% of households do not have a flush toilet; and 42% of households do not have piped water in their dwelling or inside their yard.^11^

The peri-urban study site selected is located in the Western Cape’s largest interior town 120 km northeast of Cape Town in the Du Toitskloof mountains within the Breede Valley District.^12^ The clinic provides primary health care services to a population of nearly 30,000 predominately Afrikaans speaking (90%) coloured South Africans (72%).^12^ Situated within the Cape Winelands, this peri-urban site is located in a geographic area with a population density of 1700/km^2^ characterised by low crime rates, low unemployment (14%), and access to basic services including housing. An estimated 12% of Breede Valley households are informal dwellings; 16% of households do not have a flush toilet; and 31% of households do not have piped water in their dwelling or inside their yard^11^.

In Gauteng, the urban health facility selected is in Soweto, within the City of Johannesburg Metropolitan Municipality, approximately 15 km^2^ from the city. Soweto has a total population of 1,271,628, population density of 6357 persons/km^2^ and 355, 331 households; 19% of households have no monthly income; 16% of households are considered “informal” dwellings; 8% of households do not have a flush toilet; and 45% of households do not have piped water inside their dwelling.^13^ The specific area surrounding the study site has a population density of 10,000/km^2^ and is characterised by spatial divisions of old settlements, latter-day affluent housing, and informal shack settlement.^13^ The clinic provides primary health care services to a population of over 95,000 predominately isiZulu speaking (34%) Black Africans (100%) from one surrounding area and from a second surrounding area with a population of over 8000 predominately isiXhosa speaking (47%) Black Africans (99%).^13^

### Data collection, participant recruitment, and consent

Recruitment for both sites varied as a result of COVID-19 infections and related safety protocol in clinics during the period of data collection. Data collection began in September 2021 and coincided with South Africa’s third COVID-19 wave. At this time, Gauteng province had seen the end of the third wave and the study team were allowed access to the clinics and conducted all interviews face-to-face in the antenatal care section of the MOU. At the beginning of the study, the high COVID-19 prevalence in the Western Cape did not allow for clinic visits; interviews with women in the Western Cape began telephonically (*n* = 11), and changed to site-based interviews once clinics were re-opened for data collection (*n* = 14). Based on emerging findings from the initial debriefs, the number of interviews in the Western Cape was expanded to include additional in-depth interviews with postpartum women to further enhance understanding of beneficiary preferences for onward data use.

In the Western Cape, phone numbers for remote interviews were captured by recruiters based in the study clinics and trained by the project. Lists of potential participants identified by recruiters were then provided to the study team. Interviewers then sought to call these individuals. When initial contact was made over the phone, the interviewer explained how they got the patient’s phone number and explained the purpose of the study, how long the interview would take, and informed them that they have the right to choose whether or not they wanted to proceed.

For both remote and in-person interviews, if the respondent wanted to proceed, the interviewer then read the consent form slowly and paused frequently to ask the respondent whether they understood what had been read. If the respondent indicated that they did not understand, the interviewer would reread the text and discuss it with the participant until clarified. After reading and discussing the entire consent form and answering any questions that the respondents may have, the interviewers asked the participants if they would like to proceed with the interview and allow audio recording of the interview. Those respondents who agreed to the recording were asked to say their full name, the date of the interview, and to indicate their preferences for participating, audio recording, and being quoted by saying “Yes/No” to each of these points after these sections of the consent form were read again by the interviewer.

Respondents were compensated for travel and time costs using electronic vouchers ranging in amount from R150 to 200 (US$8.00 to US $10.50) across sites depending on established precedence in each clinic. Healthcare providers were not reimbursed for participating. Telephonic interviews ranged from 60 to 90 minutes in length while in-person interviews lasted between 30 and 60 minutes in duration. Differences in length by modality were attributed to challenges with remote interviews including phone sharing and availability, poor audibility, network connectivity, or environmental circumstances. All interviews were audio recorded, translated into English where necessary and transcribed.

### Data analysis

Data were analysed through a two-step process: 1) iterative debriefs conducted concurrently with data collection and 2) through coding of transcripts using qualitative analysis software app Dedoose (https://www.dedoose.com). As part of daily debriefs, notes were entered into a Google spreadsheet organised by domains with participant responses depicted in columns. Once translated and transcribed, interviews were uploaded into Dedoose and coded by sub-domain. The codes applied to text in Dedoose were organised as “parent codes” covering the domains of the interview guides including the background of the respondent, respondent’s use of cellphones and experience providing their phone number, why patients may provide incorrect information, understandings of the word “consent”, understanding of the consent language script, respondent reflections on the consent process, and respondent views on accessing their own health data. Within each parent code, there were multiple child codes for tagging specific aspects of the text within that domain.

Results are presented according to the conceptual framing (Figure 1), wherein we first report on understandings of the word “consent” and respondent views on the current informed consent processes and we then report on preferences around how data should be used. In the discussion, we build from these findings on current practice and ideal use to propose improvements to consent processes (“what information participants need to hear”) and onward data use (“what permissions participants need to give”). Quotes and/or summary content are attributed to individual respondents using a unique code which abbreviates the province (WC for Western Cape, GP for Gauteng Province); respondent type (antenatal care patient (ANC patient) or health care provider (HCP)); native tongue language of the respondent; and in-depth interview number.

### Ethical approval

Ethical approval was obtained from institutional review boards at the University of Cape Town, Faculty of Health Sciences Human Research Ethics Committee Ref: 841/2020 dated 24 May 2021 and at the University of Witwatersrand, Human Research Ethics Committee (Medical) Ref: 201126 dated 19 July 2021.

## Results

### Sample and characteristics

Study participants included pregnant and postpartum women attending MOU services (*n* = 62), and facility providers and managers (*n* = 12). Pregnant and postpartum women had an average of 11.5 years of schooling (level of education could also be described proportionally, i.e. 60% (37/62) had completed secondary school, and 19% (12/62) had some post-secondary school education), 23% (14/62) were employed full time, and 60% (37/62) originated from outside the province where the interviews took place (Table 1). The sample population was nearly evenly split by language group as a result of requirements for the cognitive interviews conducted as part of this research and presented in a companion paper elsewhere. ^23^ An estimated 17% (11/62) were native tongue English speakers, 17% IsiZulu (11/62) speakers, 17% SeSotho (11/62) speakers, 17% Tswana (11/62) speakers, 24% IsiXhosa (15/62), and three of the additional interviews moved back and forth between English and isiXhosa. for a total of 62 maternity care respondents.

**Table 1.** Respondent sample

Respondent type	Western Cape	Gauteng	Total
MOU respondents	25	37	62
Education			
Primary or less (≤7 years)	0	1	1
Less than secondary (8–11 years)	12	12	24
Secondary (12 years)	7	18	25
Post-secondary (>12 years of school)	6	6	12
Employment status			
Employed (full time or partially)	4	10	14
Not employed / unemployed	20	22	42
Full time student	1	5	6
Healthcare providers	7	5	12
Type of provider			
Professional nurse	1	2	3
Midwife	4	1	5
Operational manager	1	0	1
Administrative Clerk	1	1	2
Research Assistant	0	1	1

### How is the word “consent” understood?

The concept of consent was familiar to both providers and patients alike and commonly understood to mean “an agreement” or “giving permission”. However, the English language word “consent” was less familiar than words like agreement or permission. A number of respondents conflated the English word “consent” with “concerned” (WC_ANC patient_English_05; WC_ANC patient_English_06). Equivalent words for consent in African languages (*tetla* in SeTswana, *tumellano* in SeSotho, *imvume* in IsiXhosa, and IsiZulu) were more commonly understood and recognised in comparison to the English word “consent”. Some respondents with higher levels of education did associate the English language word “consent” with the equivalent African language word.

### Consent language used in patient communication

Formal procedures for requesting verbal and written informed consent from patients both to perform specific procedures and to collect health data were reportedly not used in study clinics. Varied practices were reported with regard to what was communicated to patients about data collection and use, when, and by whom. Providers describe needing consent from health care clients in the context of specific tasks and primarily associated its need with the conduct of physical exams or performance of specific tests (e.g. HIV testing, sonograms, blood tests) and not for onward use of data. This variability contributed to a general feeling of beneficiary mistrust and uncertainty with regard to how they were to be used, by whom, and why the collection of those data was needed in the first place.

### Informed consent processes

#### Health data collected

##### Residential and personal information data

When patients visit a clinic for the first time, they are asked by a data clerk to present identification documents, proof of address (e.g. utility bills or bank statements), and two phone numbers on which they could be contacted. Data clerks reported asking patients only for the data, and in some instances described its need as being for purposes of recontact or reaching next of kin in the event of an adverse event, but did not report administering formal informed consent.

Perceptions of the repercussions of not providing these data (at all or inaccurately) varied. In the Western Cape, patients unable (or unwilling) to provide this information were not denied services (WC_HCP_English_07) but were told in some sites by data clerks that they would receive services only after those clients who did provide these data (WC_HCP_English_05). Some patients expressed apprehension about how these data were to be used and general confusion about why it was being collected. Healthcare providers reported that when they tried to use contact information to follow up with patients, they often found that the data were not correct and were unable to reach patients. They attributed inaccurate data to some instances of patients deliberately providing incorrect data (WC_HCP_English_01) and other instances of patients not knowing their phone number (WC_HCP_English_06, GP_HCP_isiZulu/English_05, WC_HCP_English_06), changing their SIM (WC_HCP_English_05), losing or having their phone stolen (WC_HCP_English_07, WC_HCP_English_01) and/or moving geographic residence which rendered data provided on earlier visits as being out of date. Phone sharing practices were also noted as an impediment to reaching target patients (WC_HCP_English_07).

Providers discussed two strategies to try to improve the quality of personal contact data collected about their patients. First, some providers sought to collect these data directly (rather than relying on the data clerk) so that they could communicate directly to patients about why these data were needed and how they would be used (WC_HCP_English_01) or so that they could use a confrontational approach, including scolding or shouting at patients, to solicit this information (WC_ANC patient_ENG_02). Second, some providers described engaging non-profit organisations with community health workers or law enforcement to find patients; this approach was only discussed in the Western Cape, and for cases where adverse health events necessitated contacting patients whose contact details were found to be inaccurate (WC_HCP_ENG_04).

Beneficiary perceptions about data quality concerns were challenging to glean in our study sample. This was attributed to higher levels of trust associated with our study sample given their willingness to engage with researchers in the first place. However, one study respondent (WC_ANC patient_ENG/IsiXhosa_04) did report deliberate provision of the incorrect residential information and phone number for next of kin based on concerns about HIV status disclosure. Generally, respondents expressed feeling obliged to give correct data because they associated its provision with the quality of care received and sought to avoid conflict with the healthcare providers on whom they depended for care.

##### Health status, laboratory, clinical data

Beyond the collection of residential and personal information data, we explored perceptions around the collection of personal data on health status, laboratory, and clinical tests. Within labour wards across study sites, patients were observed and reported to receive a written informed consent document which sought permission to perform specific clinic and laboratory exams within the MOU. Data capture and use were not mentioned in the form. The notion of seeking and providing explicit consent for the onward use of health data was not widely known amongst either providers or patients.

##### Photographs

Photographs were collected to support the referral, diagnosis or management of patient care. In the Western Cape, providers in the chronic disease ward, who also provided emergency services, cited examples of using their personal mobile phones to photograph injuries and seek inputs from colleagues on WhatsApp either through 1:1 chats or by posting the photographs on larger provider group chats. To support referral, the National Department of Health’s VULA app was also widely used and included a component which allowed for the uploading of patient photographs. Consent to take photographs was variably obtained, with some providers reporting soliciting verbal consent but generally no formal procedure for collecting or updating patient preferences was in place. Data on patient perceptions of photographs were not collected.

#### Current practices for health data storage, transmission, sharing

Patients understood their health data to be stored in hardcopy folders within the health facility. The concept of electronic data and aggregation of data was unfamiliar to patients. Providers understood that patient level data were aggregated and shared with the department of health for the purposes of health research, including monitoring.

In the Western Cape, the Provincial Health Data Centre aggregates some personal health data, including laboratory and pharmacy records, for use in health services research. None of the patients interviewed were aware of the data centre and potential use of their data. Among providers, awareness was limited and those providers who were aware were unable to provide specifics on how patient health data were being used, stored, or transmitted.

Photographs collected to support the provision of care were captured on the personal mobile phones of providers and a number of concerns around data sharing, access, and storage were expressed. Reliance on provider phones to capture photographs meant that patient photos were intermingled with personal photos, and potentially accessible to others with whom the phone might be shared or accessible including the provider’s children or family members. Photographs were also shared on WhatsApp or uploaded to the VULA app to support patient referral and management. While providers reported making an effort to anonymise patient features, they noted concerns about anonymisation as well as the further use and dissemination of photographs once shared.

### Patient preferences for onward health data use

Patients were asked about data use preferences for the following illustrative examples: 1) Mobile health services, including (a) sending of general health information content and (b) delivery of personalised information about pregnancy and child health via their mobile phone through text or WhatsApp; 2) Health service research, including onward use of (a) woman’s anonymised health data and (b) child’s (up until 5th birthday) anonymised health data; and 3) Use of data for recontact. Understanding of these use cases varied and was often challenging to convey to participants who understood their health data in the context solely of information inputted into their paper folders.

#### Mobile health services

##### Sending general health information

Since 2014, the National Department of Health has sought to register pregnant women attending public sector ANC services to receive general stage-based health information content as part of their flagship maternal messaging programme MomConnect. Respondents were asked about their willingness to receive “general health information about pregnancy and the baby’s health through SMS or WhatsApp”. Respondents were generally agreeable to the offer of receiving health information content over their mobile phones. Some respondents equated the receipt of these messages as being indicative of the Department of Health “caring for them”, while others acknowledged that during clinic visits “you don’t really get time to ask these questions – you are even frightened to ask these questions because [nurses] are doing things out of anger” (GP_ANC patient_SeSotho_08). Respondents overwhelmingly expressed a desire to “know more” about their health and the health of their baby (WC_ANC patient_ENG_10, WC_ANC patient_ IsiXhosa_ 07, WC_ANC patient_ IsiXhosa_02, GP_ANC patient_ SeSotho_10, GP_ANC patient_Tswana_07, GP_ANC patient_Zulu_GP) and felt that health information messages would be beneficial and “teach them about the stages child would go through” (WC_ANC patient_ENG_06).

##### Delivery of personalised health information

When asked about preferences regarding the use of health data for providing tailored health information content, including appointment alerts and reminders, respondents were similarly positive. Respondents noted that these could help them to prioritise clinic attendance. Some facilities were reportedly sending appointment alerts via text message already (WC_ANC patient_ IsiXhosa_10), however, this service was mainly for patients who were classified as high risk.

#### Health services research

##### Use of woman’s anonymised health data

Patients and providers were generally unaware that their health data were being used outside of the clinic. When asked about perceptions about the “Department of Health using women’s anonymised health data for the purposes of health research”, opinions varied. Respondents often conflated the “Department of Health” with their clinic and thus assumed the data stayed within the clinic. Many asked for clarification on what was meant by “anonymisation”. When it was explained as “having one’s name and identifying information removed,” some respondents insisted that anonymisation was not needed since they are not “hiding” anything (WC_ANC patient_English_07) and others said that only those with stigmatised health conditions (particularly HIV) would want or need anonymisation. Many respondents did not differentiate between anonymisation for onward use and anonymisation for their own health care. These respondents were therefore worried that their name would be removed from their file, resulting in their healthcare providers not knowing who they were or how to treat them. Many expressed anxiety about the loss of paper-based folders; an occurrence which could lead to a denial of health facility services or have implications for the quality of care received.

Beyond concerns about language semantics, patients were generally willing to allow for their anonymised data to be used for the purposes of health services research, indicating that it could be beneficial in “helping” or “teaching” others (WC_ANC patient_isiXhosa_10). Others felt that it could be used broadly to support added resources for care and add to a general understanding of a particular illness or health condition. Some patients, however, were unwilling to consent for use of anonymised health data, preferring instead to have their data use be restricted to that needed for their individual medical care. Others expressed reluctance out of concern about true anonymisation.

##### Use of child’s (up until 5th birthday) anonymised health data

Respondents generally asked for specificity with regard to child health data to be used, its intended uses, and the implications of that use. Some respondents understood “Child’s health information” to refer to diseases or disabilities a child might have/be born with, as opposed to information on vital statistics, health status (e.g. weight, height, head circumference), or facility contacts. Patients also were confused about the implications of anonymisation for their child’s facility-based paper folder; perceiving that anonymisation could remove important identifying information needed for future facility visits. Views on the use of child’s anonymised health data were similar to those expressed for women and generally patients were in favour of using their children’s anonymised health data for health services research. However, among those that did express reluctance, concerns were raised about true anonymisation and the need to protect their children’s information, safety, and privacy.

#### Use of data for recontact

Patients and providers were asked about their perceptions of the use of health data to support efforts by the Department of Health and partners for recontact for the purposes of joining future research studies. Some misunderstood this question to be asking them whether they wanted to join a new study at this time, rather than potentially be contacted about a new study in the future. However, overall, patients were universally supportive of being recontacted. Some suggested that these added contacts were evidence of continued care and concern: “I want to be called again … to be checked up on as to whether I am still okay” (WC_ANC patient_isiXhosa_11). Other participants (particularly those with higher levels of education) conveyed respect for research, and indicated a desire for future participation out of perceived moral obligation.

### Patient preferences for accessing health data

Patient access to health records was limited, and constrained to patients reading the various paper-based record booklets that were filled out in the clinics. In the Western Cape, patients were provided with an A4 size paper-based Maternity Care Record book. In Gauteng, patients in some clinics had maternity books and in others had a single A4 page which was folded into a pamphlet. Paper-based booklets included sections for name; residential information; chronic conditions including HIV, diabetes, and hypertension (on the front page); pregnancy history; prior birth outcomes; medication; pregnancy progress; and clinic visit dates.

When asked about preferences for electronic data, patients overwhelmingly were in favour of digitisation. They viewed electronic access to their health data as facilitating rapid and continuous access to health information: “I do not have to wait for a certain date for it to be given to me to know about what is going on now” (GP_ANC patient_SeTswana_05). Providers were similarly positive about improving patient access to health information with an emphasis on appointment dates and reminders, medications, and general information about diagnoses. Some noted concerns about patients having access to “doctors notes”, indicating that this could create some confusion (GP_HCP_English_04). However, in general, respondents felt that greater access would “give [patients] reassurance and [knowledge about] what is happening in their body and what is happening with the growth of their child” (WC_HCP_English_07). They also noted that paper-based maternity cards were kept in varied states and digital access to data could facilitate continuity of care (WC_HCP_English_03). Others equated increased access to health information with potentially reducing the need for facility visits (GP_ANC patient_SeTswana_09).

When asked about modality (paper, phone, computer) for accessing data, patients had mixed views and generally favoured a hybrid model of paper and digital access. Phone-based apps were noted for their ease of access, and lower data costs (versus websites). Theft, phone loss, and/or sharing practices were the leading concerns expressed by those reluctant to move towards mobile phone-based records (WC_ANC patient_isiXhosa_10, GP_ANC patient_ SeTswana_06, GP_ANC patient_ SeTswana_01, GP_ANC patient_ SeTswana_07). Access through a facility-based computer was seen favourably by those who expressed a desire for assistance in accessing records and with reluctance by others who felt uncertain about who at the clinic would have access to these records.

### Patient and provider preferences for improving how informed consent is captured

Providers discussed preferences around who should collect consent for health data; expressing concerns that patients might feel unable to say no if consent is obtained from the person(s) responsible for providing care. They noted the importance of having the person who solicits consent be separate from the person(s) responsible for providing medical care. Providers also generally felt that current workload demands prohibited added work to administer improved consent processes or facilitate patient-level access to electronic health records. Some indicated that administrative clerks might be best placed to assume the task of administering consent forms, while health promoters could provide group information to patients waiting for services. Patients echoed provider preferences for health promoters to provide general information about consent and consent processes. Patients suggested that they would prefer to complete forms themselves, rather than verbally stating their preferences to a staff member to record on their behalf – a factor which they felt gave them greater autonomy, and minimised pressure and judgment from providers to consent. Providers did express concerns about beneficiary literacy and language competencies, which could impede form completion.

## Discussion

The POPIA Act enacted on 1 July 2020 requires that: individuals attending public sector health services in South Africa be informed that their personal health data are being collected; outline how those data are being used and by whom; create systems for enabling data access; and allow for individual preferences to be factored into how data are processed. Study findings paint a picture of a health system with limited human resources and physical infrastructure. In this context, efforts to bolster consent practices for onward health data capture, access, and use seem like a distant priority, if one at all, to study respondents. Among patients, most were unaware that their data were being used for purposes beyond the individualised provision of medical care. The concept of anonymised use of electronic health data was at times challenging to fathom for patients, who understood their data in the limited context of paper-based folders and booklets. Improving access to health data among patients was viewed favourably by providers and patients alike; particularly an additive model which would layer digital access (via a mobile phone or a facility-based computer) atop existing paper-based folders and health booklets. Providers were observed and reported to use their personal mobile phones for a range of health data capture and exchange purposes, including facilitating referrals via the VULA app, engaging with provider groups on WhatsApp, and taking photographs to support patient care. All of these use cases represented well-intentioned efforts to improve patient care and management, but were rife with POPIA violations. Providers were aware of these tensions but in the absence of alternatives and/or clearer standards for consent capture and data sharing had few alternatives. Many providers noted concerns about who captures consent and vocalised concerns about patients’ ability to provide informed consent for onward health data use given perceived ties to care received. Future systems should endeavour to ensure that procedures for collecting onward health data consent and patient preferences do not impede the content nor quality of care received.

### Comparison with other studies

Study findings contribute to the limited literature on consent for health data collection and use as part of routine service delivery,^14–16^ particularly in low-resource settings.^17^ Our findings echo those observed elsewhere, which suggest that patients and providers alike were supportive of patient-level data use for research however, they were not well informed about the conditions or specifics of data use, sharing, or storage.^18^ A number of studies have noted concerns that providers and patients may have around the security and vulnerability of health data;^15,16,19^ provider reluctance given the time-trade-offs and existing clinical demands;^16^ harming patient-provider relations;^18,20^ and fairness in how data would be used. ^17^ In South Africa, access to and use of patient data is governed by well-established procedural and structural access controls, including data within the Western Cape’s health information exchange.^4^ However, these need to be extended to the point of data collection. Study findings on beneficiary and provider awareness of health data use should catalyse efforts to prioritise and promote consistency in onward health data capture consent procedures, including consent language, modalities, and systems for routinely collecting (and updating) beneficiary preferences.

Study findings on challenges pertaining to the use of provider personal mobile phones for a range of health data capture and exchange purposes echo those observed elsewhere in South Africa^21^ and globally.^22^ In Australia, over 50% of the 105 dermatologists and dermatology trainees surveyed across the country reported sending and receiving images on their smartphones at least weekly.^22^ Photographs were commonly stored on smartphones and shared using emails or via multi-media message.^22^ Security procedures were limited and consent documentation inadequate.^22^ Elsewhere in South Africa, a qualitative study conducted in 2014 in the province of Mpumalanga highlighted the evolving use of mobile phones among rural patients and health workers. Among providers (nurses and doctors), mobile phones were informally used for clinical emergencies, to receive laboratory results, communicate with other providers, including through WhatsApp, and to take photographs.^21^ Beyond provider frustrations with the cost implications of their phone use, concerns about consent and the implications of data capture, sharing, recontacting, and storage were not reported. Nearly a decade later, our study endeavours to build upon this evidence base and promote the need for the creation of standards to govern use of personal digital tools as part of routine service delivery.

### Implications for improving consent

This work complements a companion paper presently under peer review,^23^ which utilises cognitive testing to refine consent language for the collection and onward use of routine health data outside of personalised health care delivery. Taken together, both papers aim to improve procedures and systems for requesting informed consent for the onward use of healthcare clients’ health data for the purposes of sending health information messages, health services research, or recontact. In the context of maternity services, study findings highlight several practical learnings for the collection of health data. To improve residential and personal information data, patients need to be told more clearly how these data are to be used, by whom, and for how long. This need was highlighted by descriptions of how requests for multiple contact phone numbers caused concern to patients who were fearful of their personal health status being disclosed to family or friends. Where appropriate, patients should be given the option of providing a single number. For a number of patients, particularly those anxious about information on their personal health status being shared or known by family or community members, it is likely that issues on the quality of residential and phone numbers will persist. In such cases, non-profit organisation staff may continue to be a resource for patient follow-up at the community level. However, within health facilities, a designated counsellor with some health knowledge and training on consent, data use and access, is needed. This focal person could be drawn from existing personnel and assume responsibility for requesting informed consent for both clinical procedures and the onward use of health data; providing patients with access to their personal health records; and the means to update information and consent preferences over time.

In this study, we explored five use cases for onward health data above and beyond personalised delivery of health care: 1) Sending of general health information content; 2) Delivery of personalised information on pregnancy and child health via your mobile phone through text or WhatsApp; 3) Use of woman’s anonymised health data; 4) Use of child’s (up until fifth birthday) anonymised health data; and 5) Use of data for recontact. Selection of these was intended to capture the majority of probable use cases for personal health data, and where possible, examples were provided with each to improve clarity on what was intended and meant by each use case. While we explore efforts to hone and refine the language elsewhere, we note that for many respondents, further granularity was desired on the information about data use within these examples. As efforts gain momentum to implement improvements in the collection of consent for onward health data use, this language will need to be revisited to bolster the details being communicated to patients, including the specifics of time horizon and storage of data.

### Limitations

COVID-19 restrictions placed on the conduct of research limited the number and range of public sector facilities included in our sample. In the Western Cape sites, these restrictions also meant that many of our interviews were initially conducted remotely. Future studies may wish to include tertiary care facilities and go beyond in-depth interviews to include direct observations and focus group discussions. Study respondents were patients and providers – not those involved with the broader oversight of care, as well as the processing or use of health data. Among patients, the sample was driven by requirements for a complimentary study^23^ which sought to conduct cognitive interviews of consent language script for onward health data use in six South African languages. Accordingly, non-English speaking foreign nationals who may comprise up to 20% of MOU patients were not included. Future efforts to develop a finalised process for enhancing informed consent for onward health data use will need to solicit feedback from these populations who may be subject to marginalisation and stigmatisation.

## Conclusions

This study adds to the limited body of evidence on beneficiary and provider perceptions of how onward health data are collected and used, as well as informed consent processes, in Gauteng and the Western Cape provinces of South Africa. Future systems need to be established to collect informed consent for onward health data use. In light of perceived ties to the care received, these systems need to ensure that patient preferences do not impede the content nor quality of care received.

## Contributions

AEL, NT, KS, NP conceived the idea for the study with inputs from LF, UM, and AB. LF, UM, AB, JG, and FG acquired funding through three separate grants. AEL wrote the first draft with inputs from NT, NP, KS, and all co-authors. AEL, KS, and NP developed study tools, identified, trained, recruited, and supervised data collection. OW, KM, GM, NN, NG, MS, NM inputted on study tools, collected the data, supported analysis, and provided inputs to study findings and the manuscript. AS, LF, UM provided inputs to study design, supported patient recruitment, and provided inputs to study findings and the manuscript. All authors provided edits to the manuscript and approved the final version.

## CDC disclaimer

The findings and conclusions in this publication are those of the author(s) and do not necessarily represent the official position of the funding agencies.
